# Antibiotic Susceptibility Profiles of Dairy *Leuconostoc*, Analysis of the Genetic Basis of Atypical Resistances and Transfer of Genes *In Vitro* and in a Food Matrix

**DOI:** 10.1371/journal.pone.0145203

**Published:** 2016-01-04

**Authors:** Ana Belén Flórez, Ilenia Campedelli, Susana Delgado, Ángel Alegría, Elisa Salvetti, Giovanna E. Felis, Baltasar Mayo, Sandra Torriani

**Affiliations:** 1 Departamento de Microbiología y Bioquímica, Instituto de Productos Lácteos de Asturias (IPLA-CSIC), Villaviciosa, Asturias, Spain; 2 Dipartimento di Biotecnologie, Università degli Studi di Verona, Verona, Italy; University of Padova, Medical School, ITALY

## Abstract

In spite of a global concern on the transfer of antibiotic resistances (AR) via the food chain, limited information exists on this issue in species of *Leuconostoc* and *Weissella*, adjunct cultures used as aroma producers in fermented foods. In this work, the minimum inhibitory concentration was determined for 16 antibiotics in 34 strains of dairy origin, belonging to *Leuconostoc mesenteroides* (18), *Leuconostoc citreum* (11), *Leuconostoc lactis* (2), *Weissella hellenica* (2), and *Leuconostoc carnosum* (1). Atypical resistances were found for kanamycin (17 strains), tetracycline and chloramphenicol (two strains each), and erythromycin, clindamycin, virginiamycin, ciprofloxacin, and rifampicin (one strain each). Surprisingly, *L*. *mesenteroides* subsp. *mesenteroides* LbE16, showed resistance to four antibiotics, kanamycin, streptomycin, tetracycline and virginiamycin. PCR analysis identified *tet*(S) as responsible for tetracycline resistance in LbE16, but no gene was detected in a second tetracycline-resistant strain, *L*. *mesenteroides* subsp. *cremoris* LbT16. In *Leuconostoc mesenteroides* subsp. *dextranicum* LbE15, erythromycin and clindamycin resistant, an *erm*(B) gene was amplified. Hybridization experiments proved *erm*(B) and *tet*(S) to be associated to a plasmid of ≈35 kbp and to the chromosome of LbE15 and LbE16, respectively. The complete genome sequence of LbE15 and LbE16 was used to get further insights on the makeup and genetic organization of AR genes. Genome analysis confirmed the presence and location of *erm*(B) and *tet*(S), but genes providing tetracycline resistance in LbT16 were again not identified. In the genome of the multi-resistant strain LbE16, genes that might be involved in aminoglycoside (*aadE*, *aphA-3*, *sat4*) and virginiamycin [*vat*(E)] resistance were further found. The *erm*(B) gene but not *tet*(S) was transferred from *Leuconostoc* to *Enterococcus faecalis* both under laboratory conditions and in cheese. This study contributes to the characterization of AR in the *Leuconostoc-Weissella* group, provides evidence of the genetic basis of atypical resistances, and demonstrates the inter-species transfer of erythromycin resistance.

## Introduction

Antimicrobial agents represent one of the main therapeutic tools to protect humans and their domesticated animals from a variety of bacterial agents. However, during the last decades, the mishandling and misprescription of antibiotics in human and veterinary medicine, as well as their use as growth promoters in animal husbandry, have created a selective pressure leading to the emergence and spread of bacterial strains that no longer respond to antimicrobial therapy [1–4]. Antibiotic resistant bacteria have been largely found in soil, water, fecal material from animals and humans and in many foods of animal and plant origin, as a result of environmental contamination during processing [5].

Bacteria are said to have “intrinsic resistance” to an antibiotic when their intrinsic properties render them unsusceptible to the antibiotic’s effect. In contrast, normal susceptible bacteria may acquire resistance to an antibiotic by acquiring a new characteristic through mutation of indigenous genes or the acquisition of resistance genes by horizontal gene transfer (HGT), mostly through the transference of mobile genetic elements such as plasmids and transposons [6–8]. Particularly, the transmission of genetic material from one organism to another by HGT can greatly contribute to the dispersal of antibiotic resistances (AR), because it can occur between closely or distantly related species and in diverse environments [9–12]. Three major independent gene transfer mechanisms—namely conjugation, transduction, and transformation—are associated with HGT [13]. Among these mechanisms, conjugation is considered particularly effective at spreading of AR genes among bacteria; though it has been mostly studied under laboratory conditions [12].

While pathogens represent a direct threat to human and animal health due to their difficult eradication when carrying AR determinants, resistant non-pathogenic or opportunistic species constitute an indirect hazard, because HGT events can occur with pathogenic strains [14]. Therefore, it has been speculated that commensal bacteria can act as reservoirs of resistance genes and likely play a key role in the dissemination of AR genes in microbial ecosystems, including foodstuffs [9,15,16]. Thus, addressing the possibility of food-borne commensal bacteria being a potential source for the transfer of antimicrobial resistance genes is one issue of great importance in the field of public health.

Until now, most studies on resistant non-pathogenic species have focused mainly on some groups of lactic acid bacteria (LAB), such as enterococci, lactococci and lactobacilli [14,17]. Very limited information on the antimicrobial susceptibility profiles of *Leuconostoc* spp. is available, as well as their possible involvement in the dispersal of antimicrobial resistance determinants between bacteria.

Green vegetation and roots are considered the natural niches of *Leuconostoc*, from which they can easily propagate to the raw materials (vegetables, fruits, cereals, meat and milk) utilized in the production of fermented foods [18]. Therefore, they are frequently found as part of the natural LAB community involved in the manufacture and ripening of several fermented foods and beverages, such as kimchi, olives, meat, cacao beans, wine, pulque, and dairy products [18–20]. In dairy technology, *Leuconostoc* strains are beneficial for numerous technological aspects linked to their capacity to produce organic acids, carbon dioxide, dextrans and, especially, aromatic compounds, such as diacetyl, acetaldehyde and acetoin [18,21]. For these characteristics, well-characterized strains are intentionally added as starter or adjunct cultures in many production processes to control the fermentations and contribute to the organoleptic and rheological properties of the final product [22,23].

*Leuconostoc* strains are also linked to some negative aspects, including spoilage (such in the sugar cane industry and food products by formation of slime) and safety (they have been occasionally identified in human clinical isolates) aspects [24–26]. However, their long history of safe consumption in traditional fermented foods has led to the conclusion that *Leuconostoc* are Generally Regarded As Safe (GRAS) microorganisms. In this sense, the European Food Safety Authority (EFSA) [27] considers *Leuconostoc* to be suitable for the qualified presumption of safety (QPS) approach to their safety assessment, which requires that technological strains intended to be introduced into the food chain should lack acquired or transferable resistance determinants to antimicrobials of clinical and veterinary importance to prevent lateral spread of these [28].

The application of molecular methods, such as various PCR techniques and microarray analysis is being very helpful in determining the genetic basis of the acquired resistance phenotypes. Moreover, the recent improvements in sequencing technologies and the increasing availability of genome sequences can provide unprecedented insights into the makeup and genetic organization of AR genes [29]. To date, the complete genomes of only three antibiotic-resistant *Leuconostoc mesenteroides* strains of dairy origin have been sequenced, representing an important starting point to improve the current knowledge on the molecular basis of AR in this LAB species [30]. Thus, deeper investigations are greatly needed to examine the safety of food-borne *Leuconostoc* strains and their potential involvement in the persistence and dissemination of AR genes.

In this context, the main aims of this study were: i) to determine the antibiotic resistance/susceptibility patterns of 34 LAB strains of the *Leuconostoc-Weissella* group originating from traditional Italian and Spanish cheeses; ii) to identify the genetic basis of potentially atypical resistances encountered, assessing the presence of AR genes and their localization on the *Leuconostoc* genome; and iii) to investigate the horizontal exchange capability of specific AR from selected *Leuconostoc* strains to *Enterococcus faecalis* and *Listeria innocua*; the transferability was studied both under *in vitro* conditions and in a food matrix.

## Materials and Methods

### Bacterial strains and growth conditions

The 34 LAB analysed in this study were selected from the collections of the Department of Biotechnology of Verona University and that of Microbiology and Biochesmistry of IPLA-CSIC; they have previously been identified to the species level as *Leuconostoc mesenteroides* (*n* = 18), *Leuconostoc citreum* (11), *Leuconostoc lactis* (2), *Weissella hellenica* (2), and *Leuconostoc carnosum* (1). Strains originated mainly from the chain production of traditional Italian cheeses (Monte Veronese, Caciotta, and Taleggio) and traditional Spanish (Cabrales, Casín, and Gamonedo) cheeses (for source of the strains see Table A in S1 File). The reference strains *L*. *citreum* LMG 9849ᵀ, *L*. *mesenteroides* subsp. *cremoris* LMG 6909^T^, *L*. *mesenteroides* subsp. *dextranicum* NCFB 529ᵀ, *L*. *mesenteroides* subsp. *mesenter0oides* NCFB 523ᵀ were obtained from the BCCM/LMG Bacteria Collection, Ghent, Belgium and NCFB, National Collection of Food Bacteria (now NCIMB). Unless otherwise stated, strains were grown at 30°C in de Man Rogosa and Sharpe (MRS) broth (Fluka, Milan, Italy).

*Enterococcus faecalis* OG1RF and *Listeria innocua* LMG 11387^T^ were used as recipients in mating experiments; these were performed as reported previously [31]. The recipients and the strains used as reference for PCR detection of AR genes (see below) were cultivated at 30 or 37°C in Brain Heart Infusion (BHI) medium (Fluka).

Bacteria were kept in liquid cultures with 20% (w/vol) glycerol at -80°C for long term storage.

### Determination of phenotypic resistance

The minimum inhibitory concentration (MIC) of several antibiotics were determined according to Alegría *et al*. [21], using VetMIC (National Veterinary Institute of Sweden, Uppsala, Sweden) plates for LAB, containing serial 2-fold dilutions of 16 antibiotics (ampicillin, ciprofloxacin, clindamycin, chloramphenicol, erythromycin, gentamicin, kanamycin, linezolid, neomycin, penicillin, rifampicin, streptomycin, tetracycline, trimethoprim, vancomycin, and virginiamycin). As the concentration range of erythromycin, clindamycin, and virginiamycin in the VetMIC plates was not sufficient to measure the actual MIC to some strains, these were analysed by microdilution in Elisa plates with 2-fold dilutions of the antibiotics (obtained from Sigma-Adrich, St. Louis, Mo., USA). In addition, a mixed formulation of Iso-Sensitest medium (Oxoid, Basingstoke, United Kingdom) (90%) and MRS (10%), known as LSM [32], was used for testing tetracycline resistance phenotype of some strains.

Briefly, individual LAB colonies grown on Mueller–Hinton agar plates (Oxoid, Basingstoke, Hampshire, UK) were suspended in 2 mL sterile saline solution (Oxoid) to obtain a density corresponding to McFarland standard 1 (approx. 3×10^8^ cfu/mL). This suspension was diluted 1:1,000 in Mueller–Hinton broth (final concentration 3×10^5^ cfu/mL) and then 100 μL of this inoculum was added to each well of the VetMIC plate. Following a 48-h incubation at 30°C, MICs were visually read as the concentration at which inhibition of growth occurred.

In accordance with EFSA [27], a bacterial strain should be considered phenotypically resistant when it is not inhibited at a concentration of a specific antimicrobial equal or higher than the established microbiological breakpoint or epidemiological cut-off (ECOFF) value. However, one or two Log_2_ dilution deviations of the MICs from the cut-offs have been reported to be within the normal inter- and intra-laboratory variation in AR analyses [33].

### DNA extraction, PCR detection of AR genes and sequencing of amplicons

Total genomic DNA was extracted and purified from 2-mL overnight cultures using the Wizard Genomic DNA purification kit (Promega Corporation, Madison, USA), following the manufacturer’s instructions. Isolation of plasmid DNA was performed following the method of OʼSullivan and Klaenhammer [34] with minor modifications. Instead of the original solutions, the denaturation and neutralization steps were done by using the solutions of the commercial Plasmid Mini Kit (Qiagen, Hilden, Germany). Plasmid profiles were analysed by electrophoresis on 0.7% agarose gels in 1× TAE buffer (40 mM Tris, 20 mM acetic acid, and 1 mM EDTA), stained with ethidium bromide (0.5 mg mL^-1^), and visualized and photographed under UV light with a G. Box equipment (Syngene, Cambrigde, UK).

The presence of genes associated with resistance to erythromycin [*erm*(A), *erm*(B), *erm*(C), *msr*A], tetracycline [*tet*(K), *tet*(L), *tet*(M), *tet*(O), *tet*(S), *tet*(W)], and chloramphenicol (*cat*), was determined in the resistant strains by PCR amplification using the primers and conditions reported by Hummel *et al*. [35] and Rizzotti *et al*. [36,37] (Table B in S1 File).

For sequencing, the PCR products were purified with the Wizard SV Gel and PCR Clean-Up system according to the manufacturer’s instructions (Promega Corporation) and sent to GATC Biotech (Costance, Germany). Sequence similarity searches were performed using the BLAST network service (http://blast.ncbi.nlm.nih.gov/).

### Identification of the antibiotic resistant strains

Identification of strains at species and subspecies level was carried out using molecular biology based methods and selected phenotypic tests.

Amplification and sequencing of the 16S rRNA gene and three protein-coding genes, i.e. the genes encoding the α-subunit of ATP synthase (*atpA*), RNA polymerase α-subunit (*rpoA*) and phenylalanyl-tRNA synthase α-subunit (*pheS*), were carried out according to the indications of Rizzotti *et al*. [36] and De Buyne *et al*. [38], respectively. Primer sequences, PCR conditions and sequencing used in each case were those described by the corresponding reference (Table C in S1 File).

The *atpA* and *pheS* sequences of the isolates and type strains of species within the *Leuconostoc* genus (Table D in S1 File) were used for phylogenetic analyses using MEGA version 6 software [39].

*Leuconostoc mesenteroides* strains were tested for their ability to ferment some carbohydrates using the protocol reported by Bjorkroth and Holzapfel [19]. Briefly, strains were grown in Basal MRS-medium (pH 6.5) supplemented with a selected carbohydrate to give a concentration of 1%. After incubation at 30°C for 7 days, acid production was indicated by a change from purple to yellow in the colour of the chlorophenol red indicator dye.

### DNA hybridization

Total and plasmid DNA from erythromycin and tetracycline resistant strains was independently digested with *Pst*I, *Pst*I and *Eco*RI, and *Hin*dIII or *Pst*I, and *Nsi*I restriction enzymes (Takara, St Germain en Laye, France). After electrophoresis, the DNA was blotted onto Hybond-N nylon membranes (GE Healthcare, Buckinghamshire, UK) using a standard protocol [40]. An internal segment of the erythromycin resistance [*erm*(B)] and tetracycline resistance [tet(S)] genes, both amplified by PCR, were used as probes after labelling with Digoxigenin (Roche, Basel, Switzerland). Labelling, hybridization under high-stringency conditions, and detection was performed using the non-radioactive DIG-High Prime DNA Labelling and Detection Starter Kit II (Roche) following the manufacturer’s recommendations. AR genes were detected by chemoluminescence using an ImageQuant 350 Digital Imaging System (GE Healthcare, Pittsburgh, USA).

### Bioinformatics analysis

Sequences surrounding the AR genes from the strains *L*. *mesenteroides* LbE15, LbE16 and LbT16 were analysed by retrieving those contigs carrying antibiotic determinants from the published whole genome sequencing data [30]. The GenBank accession numbers for LbE15, LbE16, and LbT16 genomes are LAYN00000000, LAYU00000000, and LAYV00000000, respectively. The Comprehensive Antibiotic Resistance Database (CARD) at http://arpcard.mcmaster.ca/, CLC Bioinformatics Database software package (CLC bio, Aarhus, Denmark), RAST annotation system (http://rast.nmpdr.org/) and Basic Local Search Tool (http://blast.ncbi.nlm.nih.gov/Blast.cgi) were consulted for detail description of AR determinants and their flanking regions.

### Filter mating

Selected strains were included in filter mating experiments with *L*. *innocua* LMG 11387^T^ and *E*. *faecalis* OG1RF. The two recipient strains were plasmid-free and susceptible to chloramphenicol, erythromycin, and tetracycline [31]. The latter strain was further resistant to rifampicin (50 μg/mL) and fusidic acid (25 μg/mL).

Mating experiments were conducted on 0.45 μm nitrocellulose filters (25 mm diameter) (Millipore, Milan, Italy). After overnight incubation, donor and recipient cultures were mixed at a ratio of 1:10, to obtain 1×10^7^ and 1×10^8^ CFU/mL, respectively. Aliquots of the mating mixtures were filtered, and then 2 mL sterile peptone physiological solution (PPS; 0.85 g/L NaCl, 1 g/L peptone) was passed through the filter to trap the cells more tightly into the membrane. Filters were incubated over the surface of BHI agar plates without any selective agents for 24 h at 37°C. Afterwards, the filters were washed with 2 mL of PPS and the suspended bacteria were analysed by plate counting. Appropriate culture conditions were applied for separate counting of donor, recipient and tranconjugant cells. In short, MRS supplemented with 16 μg/mL tetracycline, or 8 μg/mL chloramphenicol or 4 μg/mL erythromycin was used for counting the different *Leuconostoc* donors; BHI with 50 μg/mL rifampicin plus 25 μg/mL fusidic acid was used for counting the recipient strain *E*. *faecalis* OG1RF; and Listeria Selective Agar (LSA, Oxoid) base was used for enumerating the recipient strain *L*. *innocua* LMG 11387^T^. Transconjugants of *E*. *faecalis* and *L*. *innocua* were selected on BHI agar supplemented with rifampicin and tetracycline or chloramphenicol or erythromycin, at the same concentrations reported above, or on LSA supplemented with one of the antibiotics, respectively.

Transfer frequency was expressed as the number of transconjugants per recipient.

### Food mating

Only donor and recipient strains giving transconjugants in filter mating experiments were used.

All the following procedures were performed using sterile tools under a sterile cabinet. To perform mating trails, Monte Veronese cheese slices (8 cm^3^–40 mm × 20 mm × 10 mm) were placed in Petri dishes and the surface was inoculated with a mixed culture (0.5 mL) of donor and recipient strains. Inoculum was prepared from overnight cultures that were centrifuged at 8000 × for 5 min. The pellets were washed twice with PPS, suspended in PPS and mixed to obtain 1×10^7^ and 1×10^8^ CFU/mL of donor and recipient strains, respectively. After incubation at 37°C for 24 h, the cheese slices were washed with 1 mL sterile PPS, and counts of donors, recipients and transconjugants were determined using the culture conditions reported above. Four replicates for experiment were conducted.

### Characterization of transconjugants

Presumptive transconjugants were isolated from selective agar plates and grown in BHI broth with appropriate antibiotics. To distinguish them from donor mutants, they were typed with primer Hpy1 (5’-CCGCAGCCAA-3’) using the Random Amplification of Polymorphic DNA (RAPD)-PCR technique as reported by Akopyanz *et al*. [41]. Then, transconjugants were checked for the presence the AR gene under consideration by specific PCR. Finally, the effect of such transfer on the phenotype was examined by determining the MIC of the specific antibiotic as described above.

## Results and Discussion

### Determination of phenotypic resistance

The MIC values of several antibiotics encompassing nearly all important pharmacological classes was determined by broth microdilution in VetMIC plates for 34 LAB strains belonging to the genera *Leuconostoc* (32 strains) and *Weissella* (two strains) isolated from Italian and Spanish traditional cheeses. The MICs obtained for the 16 different antibiotics and the relative ECOFF values are summarized in Table 1. To distinguish resistant from susceptible strains, the MICs were compared to the epidemiological cut-off (ECOFF) values reported by Danielsen and Wind [42], Flórez *et al*. [43], Casado Muñoz *et al*. [44] and defined according to the European Commission SCAN [45] and EFSA [27] for the genera *Lactobacillus* and *Leuconostoc*. When not defined, the breakpoint values suggested by the National Committee for Clinical Laboratory Standards [46] and Geenen *et al*. [47] for staphylococci were considered.

**Table 1:** Distribution of MICs of 16 antibiotics for LAB strains belonging to the genera *Leuconostoc* (32 strains) and *Weissella* (two strains) originated from Italian and Spanish cheese milk and dairy products.

Antibiotic		No. of isolates with the following MICs (μg/mL)										
	<1	1	2	4	8	16	32	64	128	256	512	ECOFF*(μg/mL)
Gentamycin	9	15	10									16
Kanamycin			3		8	**6**	**13**	**2**	**1**		**1**	16
Streptomycin			2	3	3	22	3		**1**^**a**^			64
Neomycin	8	5	16	3	1		1					8
Tetracycline			19	13			**1**	**1**				8
Erythromycin	31	**2**								**1**^**a**^		1
Clindamycin	33									**1**^**a**^		1
Chloramphenicol			1	**12**	**19**	**1**	**1**					4
Ampicillin	29	5										2
Penicillin G	34											1
Vancomycin										**34**^**a**^		≥ 32
Virginiamycin	25	8							**1**			4
Linezolid		2	27	5								≥ 8
Trimethoprim					**1**	**7**	**4**	**1**	**21**^**a**^			8
Rifampicin	10	8	13	**2**	**1**							≥ 4
Ciprofloxacin				15	16	2		**1**				>32

*Epidemiological cut off (ECOFF) values for strains were based on those provided by Danielsen and Wind [42], Flórez *et al*. [43], Casado Muñoz *et al*. [44], and defined according to EFSA [27], and the European Commission, SCAN [45] for the genera *Lactobacillus* and *Leuconostoc*. When not defined, the breakpoint values suggested by the CLSI [46] and Geenen *et al*. [47] for staphylococci were considered. Resistant strains with a MIC value higher than the ECOFF reported in the table are indicated in bold.

^a^These were the highest concentrations assayed; MICs of the antibiotics should be read as > the actual figure.

As expected, all analyzed strains were insensitive to high concentrations of vancomycin (MIC ≥ 128 μg/mL), since this is a common trait for species belonging to the *Leuconostoc-Weissella* group [24]. Such intrinsic characteristic is linked to the presence of D-Ala-D-Lactate in their peptidoglycan rather than a D-Ala-D-Ala dipeptide [18]. Moreover, they all were resistant to trimethoprim (MICs ≥ 8 μg/mL) for the absence of the folic acid synthesis pathway [48]. However, a broad MIC distribution (from 8 to 128 μg/mL) of this antimicrobial was observed.

In contrast, all strains were susceptible to the beta-lactams ampicillin and penicillin G, to gentamycin and linezolid (MICs lower than the microbiological ECOFFs). Some studies have previously shown that *Leuconostoc* strains isolated from dairy and meat products are susceptible to many of these antibiotics and in particular to the beta-lactams [17,49]. A broad MIC distribution characterized the remaining antibiotics, wherein we can find one or more resistant strains, belonging to different species.

Concerning aminoglycosides, most of the strains (23 out of the 34) exhibited resistance to kanamycin (MICs ≥ 16 μg/mL). The MIC distribution of kanamycin was broad, ranging from 2 to 128 μg/mL with one strain (*L*. *mesenteroides* LbE16) being resistant to more than 128 μg/mL. Kanamycin resistance was found in *L*. *mesenteroides* (9 strains), *L*. *citreum* (11), *L*. *lactis* (2) and *L*. *carnosum* (1). This observation corroborates data reported in previous studies, in which the profiles of kanamycin resistance in *Leuconostoc* spp. vary largely among strains [21,50].

MICs of the streptomycin were between 2 and 128 μg/mL, with only one strain (LbE16) being resistant to 128 μg/mL. Although this, the data obtained here suggest that the cut-off of streptomycin and kanamycin for *Leuconostoc* should be updated, for which evaluating MICs in a larger number of strains is encouraged.

MICs of neomycin were lower than the breakpoint (8 μg/mL) for all strains, except *L*. *mesenteroides* LbE16 and CA5 (32 and 8 μg/mL, respectively).

The lack of cytochrome-mediated transport is thought to be responsible for the resistance of anaerobic and facultative bacteria to aminoglycosides [51]. However, the presence of strains isolated from the same environment showing low and high MICs to aminoglycosides is largely unexplained and needs to be addressed further. High MICs may also anticipate the presence of dedicated (acquired) resistance genes [52,53]. Low rates of resistance to aminoglycosides have also been observed by Rodríguez-Alonso *et al*. [54] and Morandi *et al*. [49] for *Leuconostoc* strains isolated from artisan Galician and Italian raw milk cheeses, respectively.

All strains, except one *Weissella* strain, displayed resistance to chloramphenicol (MICs ≥ 4 μg/mL) with MICs between 4 and 32 μg/mL. Previous reports have indicated that most *Leuconostoc* species are susceptible to this broad spectrum antibiotic, since the proposed microbiological breakpoint was higher, i.e. 16–32 μg/mL [43]. The possibility of an intrinsic resistance of *Leuconostoc* species to chloramphenicol exists, which would reduce the horizontal transferability of this resistance to other bacterial species. However, this possibility cannot exclude the presence of dedicated genes providing resistance to this antibiotic, especially in the two strains displaying a high level MIC to chloramphenicol.

Concerning ciprofloxacin, a second-generation quinolone that inhibit bacterial nucleic acid synthesis, only a strain of *L*. *citreum* (CA7) was considered resistant, displaying a MIC value higher than 32 μg/mL. On the contrary, Morandi *et al*. [49] found that 83% of the 35 examined strains belonging to different species of *Leuconostoc* showed phenotypic resistance to such antimicrobial. This discrepancy could be due to the different susceptibility method used in the different antibiotic resistance surveys, disc diffusion in agar [49] versus microdilution (this work).

MICs of rifampicin were between <1 and 8 μg/mL, with three strains (*L*. *citreum* CA3 and CA6, and *L*. *lactis* CA33) which could be considered resistant (MICs ≥ 4 μg/mL). Rifampicin is a broad-spectrum antibiotic that inhibits the function of RNA polymerase in eubacteria [55]. Mutations in the gene *rpoB* encoding the RNA polymerase β-chain have been previously reported to confer resistance to rifampicin in two LAB strains, namely *L*. *mesenteroides* ATCC 8293 and *O*. *oeni* PSU-1 [56]. Whether this is the case in our strains has yet to be demonstrated.

As regards the antimicrobials belonging to the macrolide-lincosamide-streptogramin (MLS) family, all strains showed a MIC ≤ 1 μg/mL for virginiamycin, except LbE16 (MIC 128 μg/mL). This streptogramin was used for decades as an animal growth promoter; however it was banned in the European Union (EU) in 1999, because of its structural relatedness to some therapeutic antimicrobial drugs used for humans. Resistance of LAB to streptogramins, including virginiamycin, is considered less common among many other protein synthesis inhibitors [57].

Most strains displayed erythromycin MICs below or equal to the EFSAʼs cut-off (1 μg/mL) except for *L*. *mesenteroides* LbE15 which proved to be resistant to high level of this macrolide antibiotic (MIC >256 μg/mL). All examined strains showed clindamycin MICs lower than the cut-off (1 μg/mL), except again for *L*. *mesenteroides* LbE15 that was resistant to such antibiotic (MIC >256 μg/mL). As erythromycin, clindamycin belongs to the MLS phenotype, and a considerable cross-resistance with erythromycin occurs due to the overlapping ribosomal binding sites of these two antibiotics [14]. A phenotypic erythromycin resistant strain of *L*. *mesenteroides/L*. *pseudomesenteroides* isolated from the Spanish traditional blue-veined Cabrales cheese has been already reported [43], however the nature of such resistance was not investigated further. Phenotypic clindamycin resistance in Gram-positive bacteria, such as in staphylococci and enterococci, has been reported to be either constitutive or inducible. Identification of strains carrying the latter resistance type may fail by using a microdilution method [58].

Finally, tetracycline MICs ranged between 2 and 128 μg/mL and two strains (LbE16 and LbT16) grow at ≥ 32 μg/mL of tetracycline. Atypical resistance levels to tetracycline have been reported in several studies for LAB strains isolated from dairy and meat foodstuffs [5,17]. However, only two strains of *Leuconostoc* spp. with tetracycline resistance were found in independent studies where strains from beef abattoirs [59] and raw pork meat [60] were analyzed. To the best of our knowledge, this is the first report in which *Leuconostoc* with phenotypic resistance to tetracycline were detected from traditional dairy products.

It is noteworthy that several strains of *Leuconostoc* with resistance to three or more antimicrobial classes (multi-drug resistant; MDR) were identified in this work. All MDR *Leuconostoc* showed resistance to at least four antimicrobials (intrinsic and non-intrinsic), and one strain proved to be resistant to nine of them. Particularly, MDR was observed in *L*. *citreum* LE46 and in four *L*. *mesenteroides* strains, as shown in Table 2. In detail, *L*. *citreum* LE46 and *L*. *mesenteroides* Zcaf2 showed the higher chloramphenicol MIC value (32 and 16 μg/mL, respectively). *L*. *mesenteroides* LbT16 was resistant/insensitive to tetracycline, in addition to chloramphenicol, trimethoprim and vancomycin. The strains LbE15 and LbE16 showed simultaneous resistance/insensitivity to vancomycin, chloramphenicol, erythromycin, kanamycin and trimethoprim, and the first strain was further resistant to clindamycin, and the second to neomycin, streptogramin, tetracycline and virginiamicin. These findings confirmed the data of Rodríguez-Alonso *et al*. [54] and Morandi *et al*. [49] that have reported the presence of *Leuconostoc* strains resistant to multiple antibiotics in artisanal raw milk cheeses.

**Table 2:** Antibiotic resistant *Leuconostoc* strains characterized in this study.

Species	Strain	Source of isolation^a^	Fermentation of				Phenotypic antibiotic resistance (MIC μg/mL)^b^	Resistance gene(s)^c^
			Arabinose	Fructose	Sucrose	Trehalose		
*L*. *citreum*	LE46	Monte Veronese cheese	nd	nd	nd	nd	CM (32), KM (128), TM (>64)	-
*L*. *mesenteroides* subsp. *mesenteroides*	Zcaf2	Curd of Monte Veronese cheese	+	+	+	+	CM (16), KM (16), TM (32)	-
*L*. *mesenteroides* subsp. *mesenteroides*	LbE16	Taleggio cheese	+	+	+	+	CM (8), EM (1), KM (512), NM (32), SM (128), TC (>64), TM (32), VI (>8)	*aadE*, *aphA-3*, *mmr*, *sat4*, *tet*(S), *vat*(E)
*L*. *mesenteroides* subsp. *cremoris*	LbT16	Taleggio cheese	-	-	-	-	CM (4), TC (32), TM (8)	-
*L*. *mesenteroides* subsp. *dextranicum*	LbE15	Taleggio cheese	-	+	+	+	CL (>16), CM (8), EM (>8), KM (32), TM (16)	*erm*(B)

^a^ For more details see Table A in S1 File.

^b^All strains were insensitive to vancomycin (>128 μg/mL).

^c^ All the genes were detected by analysis of the genome sequence, and *tet*(S) and *erm*(B) also by PCR

CL: clindamycin; CM: chloramphenicol; EM: erythromycin; KM: kanamycin; NM: neomycin; SM: streptomycin; TC: tetracycline; TM: trimethoprim, VI: virginiamycin.

n.d.: not detected.

### Identification of the MDR leuconostocs

In order to accurately characterize the *Leuconostoc* strains (LbE15, LbE16, LbT16, LE46, Zcaf2) showing atypical AR a series of further experiments were performed. Firstly, molecular identification at the species level was carried out using amplification and sequence analysis of 16S rRNA gene, to confirm the previous analysis on the identity of the strains. Indeed, the genus *Leuconostoc* was revised in the last years with the description of novel species and subspecies, such as *L*. *myukkimchi* [61], *L*. *mesenteroides* subsp. *suionicum* [62], *L*. *gelidum* subsp. *gasicomitatum* [63], and *L*. *rapi* [64]. Since 16S rRNA gene sequence data do not allow the discrimination of the four described subspecies of *L*. *mesenteroides* (*mesenteroides*, *cremoris*, *dextranicum*, and *suionicum*), additional analysis were carried out using more divergent protein-coding genes, i.e. *atpA*, *rpoA* and *pheS* [63].

Comparative 16S rRNA gene sequence analysis confirmed that the strain LE46 belonged to the species *L*. *citreum* (99.6% sequence identity) and the other four strains to *L*. *mesenteroides* (99.9%). This was further confirmed by sequence analysis of *phe*S (accession number: KT692962). The Neighbour-joining tree of the concatenated *atpA* and *rpoA* partial gene sequences revealed low relatedness (91.5–93.6%, respectively) between our *L*. *mesenteroides* strains and the same sequences from *L*. *mesenteroides* subsp. *suionicum* LMG 11499^T^. For Neighbour-joining tree see Figure A in S1 File. Significantly higher values, in the range of 99.0–99.8%, were found with the other three *L*. *mesenteroides* subspecies, due to their close phylogenetic relationships. As DNA analysis did not give a conclusive identification, strains were classified at the subspecies level by conventional phenotypic approach based on their different capacity to ferment L-arabinose, fructose, sucrose, and threalose. The carbohydrate fermentation profiles varied among the strains, as shown in Table 2. The strain LbT16 was easily identified as *L*. *mesenteroides* subsp. *cremoris* since members of this subspecies utilize a limited number of carbohydrates [19]. The ability to ferment or not the pentose arabinose allowed the differentiation of the other strains: Zcaf2 and LbE16 were ascribed to *L*. *mesenteroides* subsp. *mesenteroides*, while LbE15, that did not utilize arabinose, was included in *L*. *mesenteroides* subsp. *dextranicum*.

### Molecular detection of resistance genes

To detect genetic determinants responsible for the resistance phenotypes observed in the strains LbE15, LbE16, LbT16, LE46, and Zcaf2, the presence of well-known structural genes associated with resistance to antibiotics which inhibit protein synthesis, such as tetracycline [*tet*(K), *tet*(L), *tet*(M), *tet*(O), *tet*(S), and *tet*(W)], erythromycin [*erm*(A), *erm*(B), *erm*(C), and *msr*A], and chloramphenicol (*cat*), was investigated by PCR amplification. All positive controls produced an amplicon of the expected size (data not shown). The results are summarized in Table 2.

The *erm*(B) gene was found only in the strain LbE15, to which it should confer its erythromycin-resistance. This gene has been shown to provide MLS resistance, coding for a methylase enzyme that modifies the 23S rRNA macrolide binding sites [7]. Neither *erm*(A) and *erm*(C) genes, coding for rRNA methylases [65], or the efflux gene *msr*A, coding for an ATP-binding transporter [65], were detected in any of all other tested strains. Among LAB, *erm*(B) is the best studied and the most widely spread gene conferring erythromycin resistance [14,17,66]. However, to our knowledge, this gene has never been described in *Leuconostoc* species.

Analysis of the tetracycline-resistant leuconostocs showed that, among the screened genes, only *tet*(S), coding for a ribosomal protection protein [67], was present in the strain LbE16. The absence of all the tested resistance determinants [*tet*(M), *tet*(O), *tet*(S), *tet*(W), *tet*(L) and *tet*(K)] in *L*. *mesenteroides* LbT16 may suggest a new mechanism of resistance which can be due either to acquired genes or to a mutation of indigenous genes [27]. Indeed, the possible presence of a false positive phenotype linked to specific growth requirements of LbT16 can be excluded, since its resistance was confirmed in different media added with tetracycline, i.e. MRS, LSM, and Mueller–Hinton broth.

The tetracycline resistance genes are largely spread among LAB and more than one gene has been reported to be present in some strains [17]. Few data are available on the abundance of *tet* genes in food-borne *Leuconostoc* strains. Two previous investigations carried out on a limited number of strains have reported the presence of the gene *tet*(S) in AR strains belonging to the species *L*. *mesenteroides* [59] and *L*. *citreum* [60] isolated from meat processing lines. In addition, Morandi *et al*. [49] found *tet* determinants in tetracycline-susceptible *Leuconostoc* strains isolated from dairy products, unveiling *tet*(M) as the most frequent gene, followed by *tet*(L) and *tet*(S). Furthermore, these authors found *tet*(L) and *tet*(M) together in two *L*. *citreum* strains, and the genes *tet*(M) and *int* (the transposon integrase gene of the Tn916/Tn1545 family) in a strain of *L*. *pseudomesenteroides*.

As regards chloramphenicol resistance, the *cat* gene could not be amplified from the genomic DNA of the five resistant strains. Therefore, the genetic basis of chloramphenicol resistance could not be determined and further research will be needed to elucidate the underlying resistance mechanism. The *cat* gene encodes a chloramphenicol acetyl transferase, and was selected because it is the commonest chloramphenicol resistance gene in LAB [68].

### Location of *erm*(B) and *tet*(S) in the *L*. *mesenteroides* genome

**Fig 1:**
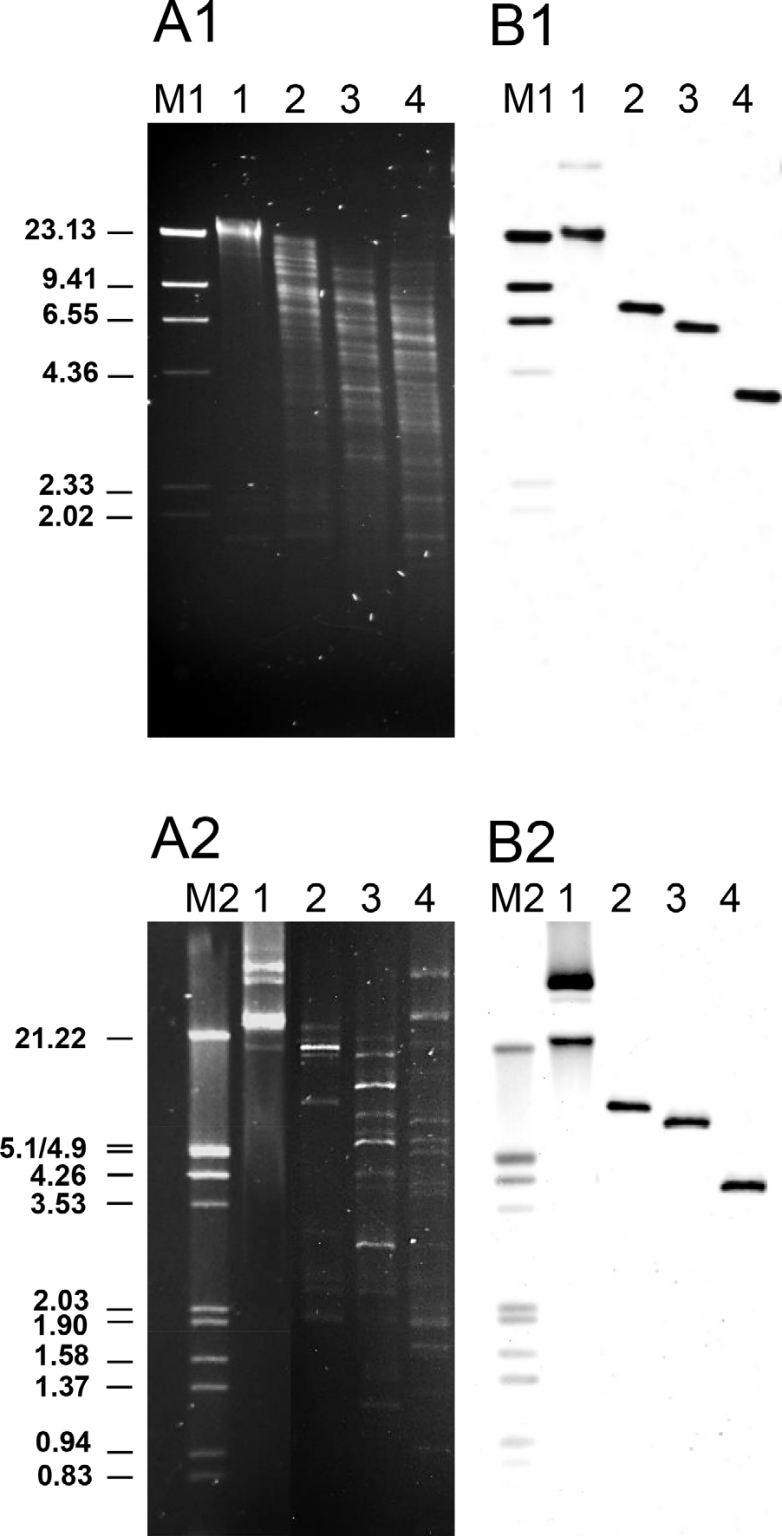
Gel electrophoresis (A) and Southern blot analysis (B) of total genomic (A1) and plasmid DNA (A2) from *L*. *mesenteroides* subsp. *dextranicum* LbE15. Lines order in the two gels: 1, undigested DNA; 2, DNA digested with *Pst*I; 3, DNA digested with *Pst*I and *Eco*RI; 4, DNA digested with *Hind*III. As a probe, an internal segment of *erm*(B) obtained by specific PCR and labelled with digoxigenin was used. M, molecular weight markers: M1, digoxigenin-labelled, *Hind*III-digested lambda DNA; M2, digoxigenin-labelled, *Eco*RI and *Hind*III-digested lambda DNA. The size of the fragments of the molecular weight markers (in kbp) is indicated.

**Fig 2:**
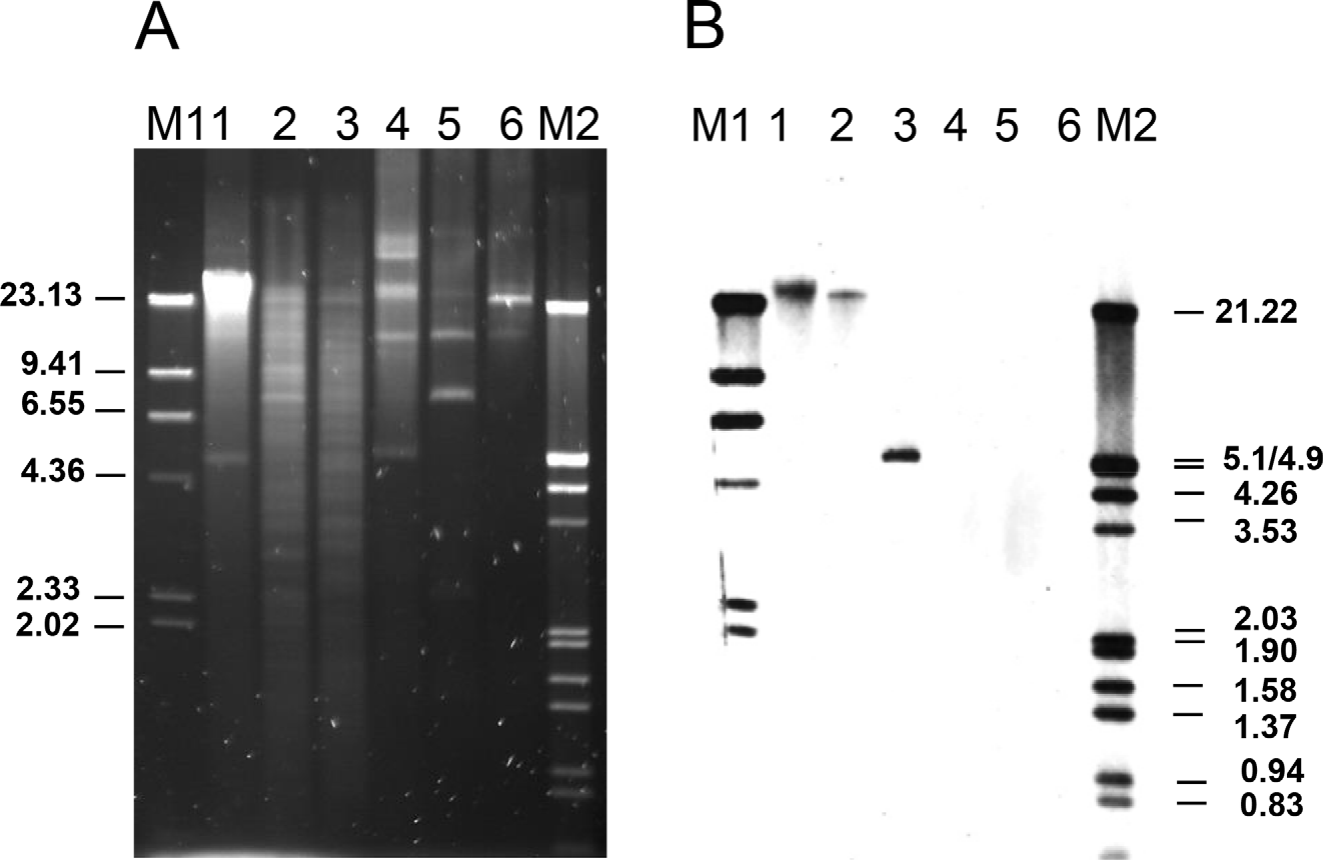
Gel electrophoresis (A) and Southern blot analysis (B) of total genomic and plasmid DNA from *L*. *mesenteroides* subsp. *mesenteroides* LbE16. Order: 1, undigested total DNA; 2, total DNA digested with (lanes 1 and 4, respectively) or digested with *Pst*I, and *Nsi*I (lanes 2, 3 or lanes 5, 6, respectively). As a probe, an internal segment of *tet*(S) obtained by specific PCR and labelled with digoxigenin was used. M, molecular weight markers: M1, digoxigenin-labelled, *Hind*III-digested lambda DNA; M2, digoxigenin-labelled, *Eco*RI and *Hind*III-digested lambda DNA. The size of the fragments of the molecular weight markers (in kbp) is indicated.

As many other LAB, *Leuconostoc* species harbour one or several plasmids of various sizes [69,70] without known functions, except for replication (cryptic). Plasmid profiling revealed at least three plasmids in each *L*. *mesenteroides* LbE15 (Fig 1, Panel A2 line 1) and LbE16 (Fig 2, Panel A line 4). Hybridization experiments using as a probe internal segments of *erm*(B) and *tet*(S), respectively, were used to identify the genetic location of these genes in the strains *L*. *mesenteroides* LbE15 and LbE16. Chemiolumiscence signals were obtained at the same positions in both the total and plasmid DNA samples from the strain *L*. *mesenteroides* LbE15 (Fig 1, Panel B1 and B2). Identical hybridization pattern of undigested total and plasmid DNA in *L*. *mesenteroides* LbE15, and also in total and plasmid digested DNA, which pointed out towards the erythromycin resistance gene linked to the largest plasmid of the strain (Fig 1). The plasmid codification of *erm*(B) leads us to suppose that *L*. *mesenteroides* LbE15 might have gained the erythromycin resistance by HGT event. On the contrary, the presence of hybridization signals in total DNA (undigested-digested) but not in plasmid DNA proved that tetracycline resistance was encoded on the bacterial chromosome of the strain *L*. *mesenteroides* LbE16 (Fig 2). Location of this gene in the *L*. *mesenteroides* genome has yet to be reported.

### Flanking regions of the AR genes

Partial sequences of the draft genome of both *L*. *mesenteroides* LbE15 and LbE16 strains [30] were used to characterize the up- and down-stream regions of the AR genes. DNA sequences from the contigs in which the AR genes were identified and the open reading frames (*orf*s) flanking the AR genes were subjected to BLASTN, BLASTX and BLASTP analyses (http://blast.ncbi.nlm.nih.gov). Genome analysis of *L*. *mesenteroides* subsp. *cremoris* LbT16 confirmed the absence of any known tetracycline resistance gene. Therefore, the high MIC displayed by this strain may be due to unspecific mechanisms, such as activity of general efflux systems, or caused by a not-yet-reported gene.

**Fig 3:**
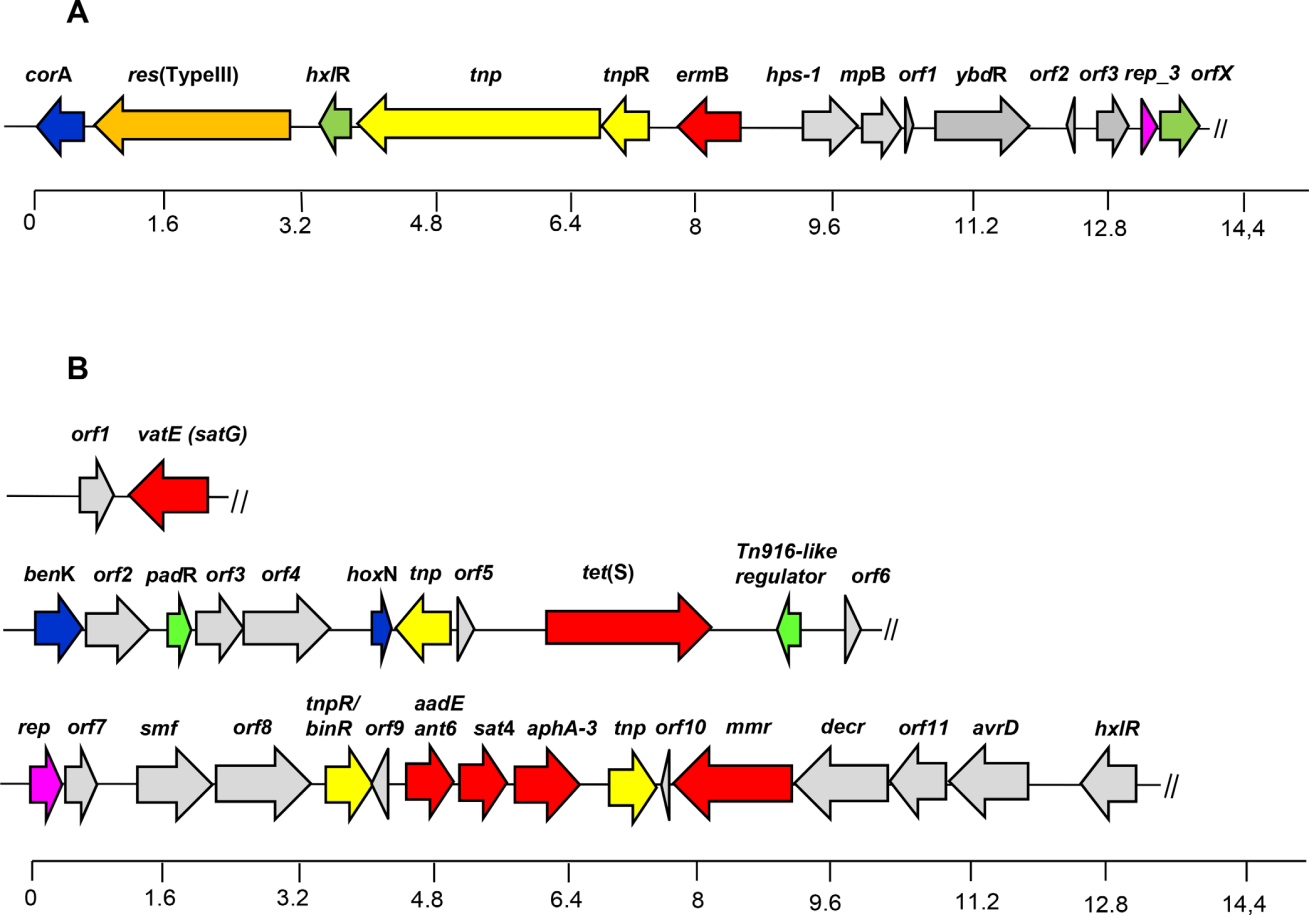
Diagram showing the genetic organization of DNA contigs around the antibiotic resistance genes identified in the genome of *L*. *mesenteroides* subsp. *dextranicum* LbE15 (A) and *L*. *mesenteroides* subsp. *mesenteroides* LbE16 (B) strains. Color code of genes and open reading frames (*orf*s): antibiotic resistance genes are in red; in yellow, genes encoding proteins involved in mobilization; in orange, genes of restriction-modification systems; in green, genes encoding regulatory proteins; in blue, genes involved in transport; in pink, genes encoding plasmid-associated replication proteins; in grey, genes belonging to other RAST subsystems. The broken line symbol indicates the end of the contig.

The AR genes found in strains LbE15 and LbE16 and their respective flanking regions are schematically depicted in Fig 3.

Only one contig carrying AR genes was detected in the *L*. *mesenteroides* LbE15 genome. This contig harboured the erythromycin resistance gene *erm*(B) detected in this strain (the *orf* in red in Fig 3A). Downstream of *erm*(B) two *orf*s encoding plasmid-replication proteins were identified; thereby supporting the association of erythromycin resistance with a plasmid, as the hybridization assays suggested. Moreover, a Type III restriction-modification system was shown to be encoded upstream of the erythromycin resistance determinant. These genes shared the greatest homology (95%) to the corresponding region of the plasmid LkipL4704 from *Leuconostoc kimchi* [71]. Furthermore, two genes encoding mobile element proteins proved to be identical at nucleotide level to those of pLG1, a plasmid of *Enterococcus faecium* [72]. These two genes shared 99% identity with the *Tn*3 DDE-transposase of several species of staphylococci, streptococci and enterococci, and the resolvase of *Streptococcus pneumoniae*, respectively [73]. Transposons are involved in the rapid adaptation of bacteria to changing environments, and their frequent location on plasmids may facilitate dissemination [74]. Therefore, the set-up of *erm*(B) flanking regions in LbE15 suggested that erythromycin resistance is an acquired character and it could be transferred among food-borne bacteria via conjugation process.

In contrast to LbE15, three contigs harbouring AR genes were identified in the genome of *L*. *mesenteroides* LbE16 (Fig 3B). The tetracycline resistance gene *tet*(S) was identified in one of the contigs. Based on the size of the contig harbouring the *tet*(S) gene (171,788 bp), it is expected that the tetracycline resistance gene is located in the bacterial chromosome of LbE16, as suggested by the Southern hybridization analysis. A small contig contained two *orf*s, of which one showed extensive homology to virginiamycin resistance [*vat*(E) in Fig 3B]. Further, a third contig harbouring a cluster of genes involved in AR was identified. These genes showed extensive homology to others involved in resistance to aminoglycosides; namely, *aadE* encoding streptomycin, *sat4* encoding resistance to streptothricin resistance, *aphA-3* encoding kanamycin and neomycin resistance and *mmr* encoding methylenomycin A resistance. The *aadE–sat4–aphA-3* cluster of LbE16 revealed almost identical nucleotide sequences with a cluster which has been previously detected in staphylococci, campylobacter and enterococci [75]. In this last contig, upstream of the streptogramin/aminoglycosides resistance genes, an *orf* that could encode a plasmid-associated protein was identified (*rep*). Moreover, the nucleotide sequence around the *rep* gene shared a complete identity with those encoded by plasmids pKLC2 [70], LkipL4726 [71] and pLCK1 [76], from *L*. *carnosum*, *L*. *kimchi* and *L*. *citreum*, respectively. This indicates that these sequences are located on a plasmid in the LbE16 strain. This cluster has also been detected in naturally occurring mobile genetic elements, such as plasmids and transposons in other bacterial species; thus is supposed to be horizontally transferable between foodborne bacteria [75]. None of the *orf*s located in the other two contigs showed significant homology to plasmid sequences, suggesting they must be chromosomally encoded. However, sequences surrounding the antibiotic resistance genes showed homology with *orf*s encoding recombinase/transposase-like proteins (in yellow in Fig 3). In particular, the sequence upstream of *tet*(S) gene showed, at aminoacidic level, 99% identity with transposase A of *Streptococcus dysgalactiae* subsp. *equisimilis* [77]. As before, these elements may contribute to the horizontal transfer of these antibiotic resistances.

### Filter mating experiments

To investigate the transferability of AR, filter mating trials were conducted *in vitro* using the three sequenced AR *Leuconostoc* strains as donors and *E*. *faecalis* OG1RF and *L*. *innocua* LMG 11387^T^ as recipients. Both these recipient strains have been shown to be susceptible to erythromycin (MIC 1 μg/mL) and tetracycline (MIC 1 μg/mL) and plasmid free [31]. Further, both recipients have already been used successfully in previous mating studies involving enterococci [31,78].

Transfer of AR genes to *L*. *innocua* LMG 11387^T^ was never achieved. However, transconjugants were obtained in the conjugation between *L*. *mesenteroides* subsp. *dextranicum* LbE15 and the recipient *E*. *faecalis* strain. Transfer was low, but detectable, with an average frequency of 3.2 × 10^−8^ transconjugants per recipient. All presumptive transconjugants, grown onto plates containing the selective antibiotics (erythromycin plus rifampicin), were isolated from each mating experiment and subjected to RAPD-PCR fingerprinting using the primer *Hpy*1 to exclude the presence of mutant donors. They displayed the same RAPD-PCR profile as the recipient strain *E*. *faecalis* OG1RF, thus confirming that they were true transconjugants and not reverted mutants (data not shown). The transconjugants displayed increased average MIC values of > 64 (erythromycin) in comparison to the original recipient MIC of 1 μg/mL. Thereafter, transfer of the genes *erm*(B) to transconjugants was verified by specific PCR, since they were selected during the experiments by their resistance phenotype. Results revealed that this genetic determinant could be PCR amplified from the transconjugants, whereas amplification was negative when DNA from *E*. *faecalis* OG1RF was used as a template (data not shown). Further, sequence analysis of a *erm*(B) gene fragment (549 pb) from donor and selected transconjugants showed, as expected, 100% identity. These findings demonstrated that transfer of the *erm*(B) gene and its associated phenotype between *L*. *mesenteroides* and enterococci can occur in laboratory conditions.

Previous studies have reported the *in vitro* transfer of *erm*(B) from different LAB species, such as *Lactobacillus fermentum*, *Lactococcus lactis*, *Lactobacillus reuteri* and *Lactobacillus salivarius*, to enterococci and lactococci [79–82]. However, until now, no successful conjugal transfer has been described for *Leuconostoc* strains. Indeed, to our knowledge, the only previous study of Toomey *et al*. [59] did not obtain transconjugants when attempting to transfer tetracycline resistance from *L*. *mesenteroides* strains harbouring *tet*(S).

### Food mating experiments

Since the laboratory transfer assays do not mimic the *in vivo* conditions, mating trials were also conducted in food using the same donor and recipient strains as above. The mating experiment was done onto the surface of Monte Veronese cheese. Twenty presumptive transconjugants were obtained, which were verified as before. An estimated conjugation frequency of around 2.2 × 10^−7^ per recipient was calculated, which was considerably higher (up to ∼16,000-fold) than that seen under standard filter mating conditions.

The present study demonstrates that HGT events can be realized in a food matrix, and that *Leuconostoc* strains could represent potential vectors of AR genes in dairy products. The possibility of transfer of AR from commensal food-borne bacteria has been studied extensively in laboratory conditions, but only a limited number of researches have been conducted in real food matrices [12]. Furthermore, almost all these investigations have considered meat-based foods as environmental niches for HGT among bacteria, especially enterococci [31,78,83]. In this context, the results of the present study appear of relevance, as the transmission of AR gene between *Leuconostoc* and *E*. *faecalis* was shown for the first time in filter mating experiments, under ideal conditions, and in a complex ecosystem, like that of the cheese. In addition, it was observed that the frequency of the transfer events found in Monte Veronese cheese was higher than those found in laboratory media; these data are in accordance with Davies and Davies [84] who suggested that frequencies of conjugative transmission in nature are probably some orders of magnitude higher than those under laboratory conditions.

## Conclusions

Resistance to different antibiotics was detected among strains of the *Leuconostoc-Weissella* group isolated from traditional Italian and Spanish cheeses. Some resistances, such as those to vancomycin, chloramphenicol and trimethoprim are—or can be- indicative of intrinsic nature, suggesting the need of future evaluation of MICs in a larger number of *Leuconostoc* strains. However, resistances of a reasonable acquired origin were also found. As such, a correlation between atypical erythromycin and tetracycline resistance and the presence of *erm*(B) and *tet*(S) genes, respectively, was encountered. The genetic basis and associated resistance mechanisms toward some other antibiotics could not be determined and would require further investigation. The genes *erm*(B) and *tet*(S) were localized on a plasmid and on the chromosome of LbE15 and LbE16, respectively. Further insights on the AR make up and the genetic organization of the AR genes were achieved analyzing the whole genome sequence of three resistant strains (LbE15, LbE16, and LbT16). Genome analysis confirmed the presence of the genes *erm*(B) and *tet*(S) in the strains in which they were previously detected and identified others encoding uncommon AR in LAB. Analysis of the genes and their flanking regions revealed the potential of some determinants to be horizontally transferred. Indeed, the data presented in this paper provide the first evidence of the erythromycin resistance transfer by conjugation between *L*. *mesenteroides* and *E*. *faecalis* both *in vitro* and in cheese, supplying novel proof that AR LAB can act as a reservoir of acquired AR genes.

## Supporting Information

S1 File Source of isolation of the *Leuconostoc*-*Weissella* strains of this study (Table A). Primers and positive control strains used for the detection of antibiotic resistant genes (Table B). Primers and conditions used for the identification of dairy *Leuconostoc*-*Weissella* (Table C). GenBank accession numbers for nucleotide sequences of the genes *atpA* and *pheS* used for the phylogenetic analyses (Table D). Phylogenetic tree obtained from the concatenated *atp*A and *phe*S gene sequences of the four *Leuconostoc* strains showing atypical AR profiles and 12 *Leuconostoc* type strains with *L*. *fallax* LMG 13177^T^ as an outgroup. The tree was reconstructed by using maximum composite likelihood method. Bootstrap values (1,000 replicates) are shown as a percentage at the branching points. The scale bar represents the number of nucleotide substitutions per site (**Fig A**).
(DOCX)
