# Supplementary material for: Antibiotic Susceptibility Profiles of Dairy Leuconostoc, Analysis of the Genetic Basis of Atypical Resistances and Transfer of Genes In Vitro and in a Food Matrix
Source: PLoS One. 2016 Jan 4;11(1):e0145203. doi: 10.1371/journal.pone.0145203 (PMC4699710; doi:10.1371/journal.pone.0145203)
Supplement: S1 File — Source of isolation of the Leuconostoc-Weissella strains of this study (Table A). Primers and positive control strains used for the detection of antibiotic resistant genes (Table B). Primers and conditions used for the identification of dairy Leuconostoc-Weissella (Table C). GenBank accession numbers for nucleotide sequences of the genes atpA and pheS used for the phylogenetic analyses (Table D). Phylogenetic tree obtained from the concatenated atpA and pheS gene sequences of the four Leuconostoc strains showing atypical AR profiles and 12 Leuconostoc type strains with L. fallax LMG 13177T as an outgroup. The tree was reconstructed by using maximum composite likelihood method. Bootstrap values (1,000 replicates) are shown as a percentage at the branching points. The scale bar represents the number of nucleotide substitutions per site (Fig A). (DOCX) [file pone.0145203.s001.docx]

**Table A. Source of isolation of the *Leuconostoc*-*Weissella* strains of this study.**

| Source of isolation | Producer | LAB species | Strain | Details of isolation |
| --- | --- | --- | --- | --- |
| (details of the cheese) |  |  |  |  |
| Monte Veronese cheese (PDO^a^ cheese from raw cows’ milk, northern Italy) | A | *L. citreum* | ZF15-4 | 15 day-old cheese |
|  |  |  | LE36 | 60 day-old cheese |
|  |  |  | LE46 | 120 day-old cheese |
|  |  | *L. carnosum* | ZLM1 | Cheese milk |
|  |  | *L. mesenteroides* | Zcaf2 | Curd |
|  |  |  | ZF30-4 | 30 day-old cheese |
|  |  |  | LE50 | 120 day-old cheese |
|  | B |  | RLM4 | Cheese milk |
|  |  |  | Rcaf2 | Curd |
|  |  |  | RF15-2 | 15 day-old cheese |
|  |  |  | LE30 | 30 day-old cheese |
|  |  |  | RF60-1 | 60 day-old cheese |
|  |  |  |  |  |
| Taleggio cheese | C |  | LbE15, LbE16 | Ripened cheese |
| (PDO cheese from raw or pasteurized cows’ milk, northern Italy) | D |  | LCT26a, LbT16 | Ripened cheese |
|  |  |  |  |  |
| Caciotta cheese | D |  | LCT23, LCT25 | Ripened cheese |
| (pasteurized cows’ milk cheese, northeast Italy) |  |  |  |  |
|  |  |  |  |  |
| Taleggio cheese | E | *W. hellenica* | LCT10, LCT11 | Ripened cheese |
|  |  |  |  |  |
| Cabrales cheese | A | *L. mesenteroides* | 3AC2 | Curd |
| (raw-milk, blue-veined, |  |  | 3AC16 | 15-day old cheese |
| traditional PDO cheese, | B | *L. citreum* | 4AC4 | Curd |
| Northern Spain) |  |  | 4AC15 | 15-day old cheese |
|  |  | *L. lactis* | 4AB2 | Curd |
|  |  |  |  |  |
| Casín cheese | A | *L. mesenteroides* | CA2, CA5 | Curd |
| (raw-milk, acid-coagulated, |  | *L. citreum* | CA3 | Curd |
| traditional PDO cheese, |  |  | CA6, CA7 | 7-day old cheese |
| Northern Spain) |  | *L. lactis* | CA33 | 30-day old cheese |
|  |  |  |  |  |
| Gamonedo cheese | A | *L. citreum* | GA3, GA5 | Curd |
| (raw-milk, blue-veined, smoky, traditional PDO cheese, Northern Spain) |  |  | GA22 | 30-day old cheese |

^a^PDO, protected designation of origin status.

**Table B. Primers and positive control strains used for the detection of antibiotic resistant genes.**

| Target gene | Primer name | Sequence (5’-3’) | Amplicon size (bp)^a^ | Positive control strain |
| --- | --- | --- | --- | --- |
| *erm*(A) | ermA-I | TCT AAA AAG CAT GTA AAA GAA | 645 | *Streptococcus pyogenes* 190*^b^* |
|  | ermA-II | CTT CGA TAG TTT ATT AAT ATT AGT |  |  |
| *erm*(B) | ermB-I | GAA AAG GTA CTC AAC CAA ATA | 639 | *S. pyogenes* C61 |
|  | ermB-II | AGT AAC GGT ACT TAA ATT GTT TAC |  |  |
| *erm*(C) | ermC-I | TCA AAA CAT AAT ATA GAT AAA | 642 | *S. epidermidis* DST-ST12 |
|  | ermC-II | GCT AAT ATT GTT TAA ATC GTC AAT |  |  |
| *mrs*A | mrsA-I | GCA AAT GGT GTA GGT AAG ACA ACT | 399 | *E. faecium* FAIR-E 349 |
|  | mrsA-II | ATC ATG TGA TGT AAA CAA AAT |  |  |
| *tet*(K) | tetK-1 | TCG ATA GGA ACA GCA GTA | 169 | *S. epidermidis* DST-SE20 |
|  | tetK-2 | CAG CAG ATC CTA CTC CTT |  |  |
| *tet*(L) | tetL-1 | ATA AAT TGT TTC GGG TCG GTA AT | 1,077 | *E. faecalis* DST-ET10 |
|  | tetL-2 | AAC CAG CCA ACT AAT GAC AAT GAT |  |  |
| *tet*(M) | tetM-1 | GTG GAC AAA GGT ACA ACG AG | 406 | *Staphylococcus epidermidis* DST-ST11 |
|  | tetM-2 | CGG TAA AGT TCG TCA CAC AC |  |  |
| *tet*(O) | tetO-1 | AAC TTA GGC ATT CTG GCT CAC | 515 | *Enterococcus faecalis* Jtet*^b^* |
|  | tetO-2 | TCC CAC TGC TCC ATA TCG TCA |  |  |
| *tet*(S) | tetS-1 | CAT AGA CAA GCC GTT GAC C | 669 | *E. gallinarum* DST-ET14 |
|  | tetS-2 | ATG TTT TTG GAA CGC CAG AG |  |  |
| *tet*(W) | tetW-1 | GAG AGC CTG CTA TAT GCC AGC | 168 | *B. animalis* subsp. *lactis* DST-Bl |
|  | tetW-2 | GGG CGT ATC CAC AAT GTT AAC |  |  |
| *cat* | Entcatfw | ATG ACT TTT AAT ATT ATT RAW TT | 540 | *E. faecalis* FAIR-E 279 |
|  | Entcatrev | TCA TYT ACM YTA TSA ATT ATA T |  |  |

^a^The PCR amplification was performed using conditions reported by Hummel *et al*. [35] for the gene *cat*, and Rizzotti *et al*. [36,37] for the genes *erm*(A)-*erm*(C) and *tet*(K)-*tet*(W), respectively.

**Table C. Primers and conditions used for the identification of dairy *Leuconostoc*-*Weissella*.**

| Target gene | Primer name | Sequence (5’-3’) | Amplicon size (bp) | Reference for primers and PCR conditions |
| --- | --- | --- | --- | --- |
| 16S rRNA | Lac16S-f | AAT GAG AGT TTG ATC CTG GCT | 1,535 | [36] |
|  | Lac16S-r | GAG GTG ATC CAG CCG CAG GTT |  |  |
| *atpA* | atpA-20-F | TAY RTY GGK GAY GGD ATY GC | 1,011 | [38] |
|  | atpA-26-R | TTC ATB GCY TTR ATY TGN GC |  |  |
| *rpoA* | rpoA-21-F | ATG ATY GAR TTT GAA AAA CC | 803 | [38] |
|  | rpoA-23-R | ACH GTR TTR ATD CCD GCR CG |  |  |
| *pheS* | pheS-21-F | CAY CCN GCH CGY GAY ATG C | 411 | [38] |
|  | pheS-23-R | GGR TGR ACC ATV CCN GCH CC |  |  |

**Table D. GenBank accession numbers for nucleotide sequences of the genes *atpA* and *pheS* used for the phylogenetic analyses.**

| Species | Strain^a^ | Accession number | |
| --- | --- | --- | --- |
|  |  | ***atpA*** | ***pheS*** |
| *L. carnosum* | LMG 23898^T^ | AM711275 | AM711282 |
| *L. citreum* | LMG 9849^T^ | AM711202 | AM711152 |
| *L. fallax* | LMG 13177^T^ | AM711284 | AM711193 |
| *L. gelidum* | LMG 18297^T^ | AM711204 | AM711160 |
| *L. holzapfelii* | LMG 23990^T^ | AM711273 | AM711209 |
| *L. inhae* | LMG 22919^T^ | AM711190 | AM711167 |
| *L. kimchii* | LMG 23787^T^ | AM711220 | AM711195 |
| *L. lactis* | LMG 8894^T^ | AM711253 | AM711267 |
| *L. mesenteroides* subsp. *cremoris* | LMG 6909^T^ | AM711203 | AM711159 |
| *L. mesenteroides* subsp. *dextranicum* | LMG 6908^T^ | AM711185 | AM711155 |
| *L. mesenteroides* subsp. *mesenteroides* | LMG 6893^T^ | AM711176 | AM711145 |
| *L. mesenteroides* subsp. *suionicum* | LMG 11499^T^ | AM711177 | AM711198 |
| *L. mesenteroides* | Zcaf2 | KT692960 | KT692961 |
| *L. pseudomesenteroides* | LMG 11482^T^ | AM711175 | AM711197 |

^a^The *atpA* and *pheS* sequences of *L. mesenteroides* LbE15, LbE16, and LbT16 were obtained from their available genome sequences (LAYN00000000, LAYU00000000, and LAYV00000000, respectively).

**
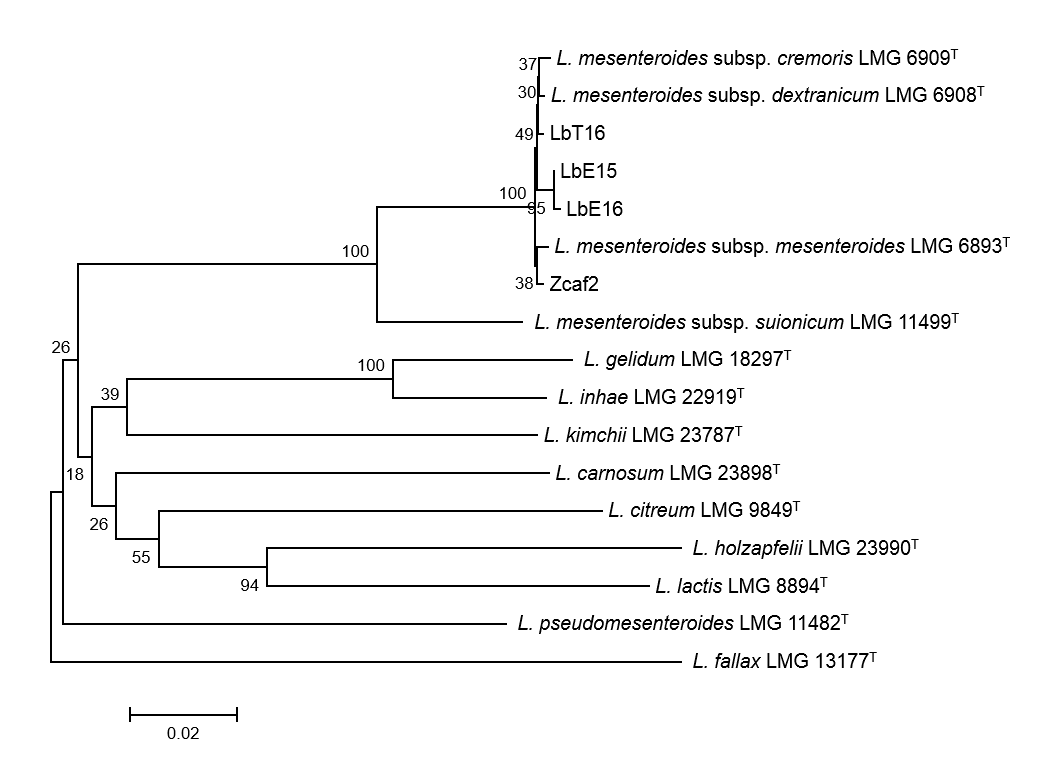
**

**Fig A. Phylogenetic tree obtained from the concatenated *atp*A and *phe*S gene sequences of the four *Leuconostoc* strains showing atypical AR profiles and 12 *Leuconostoc* type strains with *L. fallax* LMG 13177^T^ as an outgroup.** The tree was reconstructed by using maximum composite likelihood method. Bootstrap values (1,000 replicates) are shown as a percentage at the branching points. The scale bar represents the number of nucleotide substitutions per site.
